# Poly(arylene ether)s-Based Polymeric Membranes Applied for Water Purification in Harsh Environment Conditions: A Mini-Review

**DOI:** 10.3390/polym15234527

**Published:** 2023-11-25

**Authors:** Mengxue Wang, Lingsha Li, Haipeng Yan, Xidi Liu, Kui Li, Ying Li, Yong You, Xulin Yang, Huijin Song, Pan Wang

**Affiliations:** 1 School of Mechanical Engineering, Chengdu University, Chengdu 610106, China; wangmengxue@stu.cdu.edu.cn (M.W.); lilingsha@stu.cdu.edu.cn (L.L.); yhp5192022@163.com (H.Y.); liuxidi@stu.cdu.edu.cn (X.L.); leekingue2014@163.com (K.L.); liying@cdu.edu.cn (Y.L.); yangxulin@cdu.edu.cn (X.Y.); shj1437@163.com (H.S.); 2 Key Laboratory of General Chemistry of the National Ethnic Affairs Commission, School of Chemistry and Environment, Southwest Minzu University, Chengdu 610041, China; youyong@swun.edu.cn

**Keywords:** poly(arylene ether)s, polymeric membranes, water purification, harsh environments, high-performance application

## Abstract

Confronting the pressing challenge of freshwater scarcity, polymeric membrane-based water treatment technology has emerged as an essential and effective approach. Poly(arylene ether)s (PAEs) polymers, a class of high-performance engineering thermoplastics, have garnered attention in recent decades as promising membrane materials for advanced water treatment approaches. The PAE-Based membranes are employed to resist the shortages of most common polymeric membranes, such as chemical instability, structural damage, membrane fouling, and shortened lifespan when deployed in harsh environments, owing to their excellent comprehensive performance. This article presents the advancements in the research of several typical PAEs, including poly(ether ether ketone) (PEEK), polyethersulfone (PES), and poly(arylene ether nitrile) (PEN). Techniques for membrane formation, modification strategies, and applications in water treatment have been reviewed. The applications encompass processes for oil/water separation, desalination, and wastewater treatment, which involve the removal of heavy metal ions, dyes, oils, and other organic pollutants. The commendable performance of these membranes has been summarized in terms of corrosion resistance, high-temperature resistance, anti-fouling properties, and durability in challenging environments. In addition, several recommendations for further research aimed at developing efficient and robust PAE-based membranes are proposed.

## 1. Introduction

Given the rising global population and accelerated industrialization, the demand for water resources is increasing while freshwater availability remains limited [1,2]. It has become vital to develop and implement effective water treatment technologies to purify seawater and reclaimed industrial, municipal, and agricultural wastewater to ensure the availability of usable water resources [3]. Scientific advancements have led to the development of various effective water purification methods, including gravity sedimentation, coagulation, flocculation, distillation, evaporation, adsorption, filtration, ion exchange, biological treatment, etc. [4,5,6]. Currently, membrane-based technology offers prospects of efficiency and sustainability due to its ability to reduce complex equipment and high energy requirements, as well as its ease of handling and high efficiency [7,8]. However, the widespread application of membrane-based technology increases the need for high-performance core membrane materials. This arises from the extreme conditions in certain water treatment environments, such as organic solvents in oily wastewater, corrosive substances, organic pollutants in industrial wastewater, high temperatures condensed water, and biological contaminants in overall wastewater. Under these harsh conditions, inappropriate and fragile membranes make them susceptible to corrosion and contamination. This, in turn, results in structural damage, reduced efficiency, shortened lifespan, and the potential for secondary pollution caused by the decomposition of a membrane [9,10]. Hence, investigating membrane materials with tolerance to withstand such harsh environments is a pathway to make the technology more adaptable, efficient, and cost-effective.

Conventional membranes used in water treatment can be roughly classified into organic and inorganic. Organic membranes specifically refer to membranes constructed via natural polymers and synthetic polymers, also called polymeric membranes [4]. Compared to inorganic membranes, polymeric membranes are more favored for water treatment with widespread applications for several reasons. Primarily, polymeric membranes possess an apparent mechanism for pore formation with high efficiency while operating in microfiltration (MF), nanofiltration (NF), ultrafiltration (UF), and reverse osmosis (RO). Moreover, polymeric membranes offer a significant cost advantage while occupying less space and equipment during operation [4,11]. The high flexibility, diverse functional features, and facile modifiable properties of polymeric membranes also create vast possibilities for innovative applications and expand the realm of imagination in their development [12]. However, in practice, polymeric membranes do possess certain disadvantages, particularly in terms of chemical stability and mechanical performance, which are comparatively inferior to those of inorganic membranes. To overcome the above challenges, it is crucial to focus on developing robust polymeric membranes. This is usually achieved by exploring innovative core polymer materials with comprehensive performance and conducting thorough research on advanced material modification strategies.

Poly(arylene ether)s (PAEs) polymers belong to a category of high-performance engineering plastic that is widely used in various industrial fields, including aerospace, electronics, automotive, and chemical industries [13,14]. This type of polymer possesses a main chain structure featuring alternating rigid aromatic rings and flexible ether bonds, enabling them to exhibit processability as well as high-temperature resistance, acid and alkali corrosion resistance, good mechanical properties, oxidative stability, and solvent resistance [14,15]. The pioneering synthesis of high molecular weight PAEs and their corresponding commercial products can be traced back to the 1960s, and then, after more than half a century of development, the PAE family now encompasses a diverse range of members [16,17]. Among them, PAE polymers that are widely studied today can be primarily classified into the following typical kinds based on different functional groups: poly(arylene ether ketone) (PAEK) containing ketone segments in the mainchain, poly(arylene ether sulfone) (PAES) containing sulfone segments in the mainchain, and poly(arylene ether nitrile) (PAEN) pending cyano groups on the sidechain [18]. Nowadays, their applications have also been extended from engineering a structural plastics area to functional components in high-tech fields. Among them, after extensive studies in recent decades, PAEs can be prepared as polymeric membranes via phase separation, melt spinning, or electrospinning technology and are further used in the water treatment field with advanced properties [19]. Specifically, PAE-based polymeric membranes typically exhibit stable operation performance at a high temperature since PAE polymers have glass transition temperatures over 150 °C [20]. Heat resistance is an important requirement that cannot be ignored for membrane materials in water treatment, as high-temperature water flow or steam is often involved in the water treatment process. If the membrane material does not have sufficient heat resistance, it may deform, break, or lose its filtration function, affecting water treatment’s effectiveness. PAE polymers have excellent heat resistance and can maintain a stable structure and performance at high temperatures, making them very suitable as membrane materials in water treatment. They can maintain stable filtration efficiency and durability at high temperatures, ensuring the water treatment systems’ stable operation and long-term reliability [21,22,23]. Therefore, heat resistance is one of the critical factors influencing the performance of membrane materials when operating in a severe high-temperature water treatment environment. For most PAE-based polymeric membranes, they can also withstand acid and alkali corrosion as well as organic solvents and oxidizing agents (such as chlorine) [24,25,26,27,28,29], even containing a moderate amount of a polar group (-COOH, -SO_3_H, -NH_2_). According to the PAE’s good mechanical toughness and flexibility, the membrane usually demonstrates good durability [30]. Furthermore, due to the facile modifiable structure of the polymer and the advantages of the polymeric-matrix for preparing composition materials, the PAE membrane can be further multifunctionalized to resist contamination from metal ions, microorganisms, bacteria, and other pollutants [4,31]. Hence, PAE-based polymeric membranes have emerged as potential candidates for generating high-performance membranes to meet the requirements of applications in harsh environments.

This mini-review briefly outlines the development of PAE-based polymeric membranes for water treatment, centering on the typical PAE polymers: PEEK, PES, and PEN. The primary strategies for polymeric formation and functional modification have been reviewed. Relatively more focus has been given to the specific performance of PAE-based membranes that can withstand various harsh operating conditions. Then, challenges and potential future research directions will also be presented.

## 2. PAE-Based Membranes Applied for Water Purification in Harsh Environment Conditions

### 2.1. Poly(Ether Ether Ketone)

**Figure 1:**
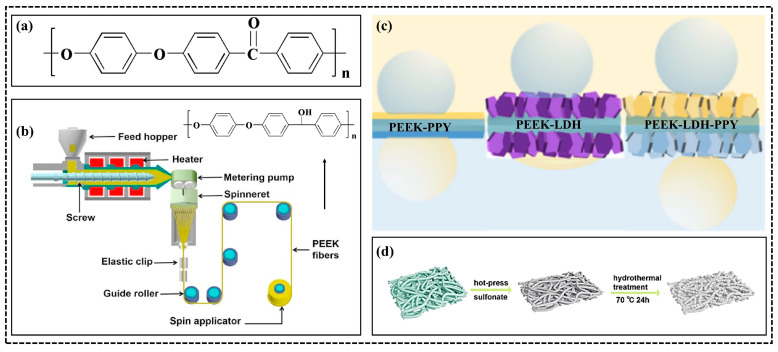
Typical molecular structure of PEEK (**a**) [32], melting preparation process of PEEK membrane (**b**) [19], double superhydrophobicity and good switching stability of PEEK membranes (**c**) [37], and diagrammatic sketch of the preparation for the ZnO nanoneedle-modified PEEK fiber felt (**d**) [38].

The PAEK family, a group of thermoplastic polymers, comprises one or more ether and ketone bonds connected by semi-crystalline aromatic moieties [32]. According to the order of an ether bond, ketone, and benzene ring connection on the molecular chain, different proportions of the polymers can be formed. Specifically, polyether ether ketone (PEEK), polyether ketone (PEK), polyether ketone ketone (PEKK), polyether ether ketone ketone (PEEKK), polyether ketone ether ketone ketone (PEKEKK), and so on. Among them, PEEK holds the distinction of being the first commercially available PAEK polymer and has since gained widespread adoption. As the typical structural formula shown in Figure 1a, the PEEK main chain structure contains ketone bonds alternating with two ether bonds, which contribute a high-temperature resistance, chemical corrosion resistance, as well as high strength and ease of process [33]. Consequently, PEEK finds diverse applications in various industries, including the chemical industry, electronics and electrical appliances, machinery and instrumentation, automotive industry, and water treatment [34,35]. Its exceptional heat resistance and outstanding performance make it a highly sought-after thermoplastic resin, particularly as a matrix material for high-performance composites. This versatility has resulted in widespread use and recognition. Since then, in the field of water purification, PEEK has become representative of the PAEK family due to its excellent performance, serving as an ideal matrix material for high-performance composites and further employed as a water treatment product, including filter cartridges, filter plates, films, and other forms. These products are specifically utilized for water filtration and separation in harsh environments like seawater desalination, wastewater treatment, etc. At present, the primary methods for preparing PEEK separation membranes are non-solvent-induced phase transition (NIPS) and thermally induced phase separation (TIPS), with post-treatment techniques such as extraction and surface grafting employed to construct porous structures suitable for the challenging conditions encountered in harsh water treatment applications [36].

#### 2.1.1. Toughness PEEK Membranes Applied in Corrosive Environments

The rigid molecular structure of PEEK membranes endows them with excellent chemical resistance and high-temperature tolerance, setting them apart in the realm of oil/water separation membranes. Moreover, the rigid molecular structure also results in a naturally hydrophobic surface of PEEK membrane materials. To enhance treatment efficiency, PEEK membrane materials are often functionalized via grafting hydrophilic groups such as –OH or –COOH [19,39] onto their surfaces, or they are prepared into hollow fibers to increase their permeation flux [40,41,42]. However, the introduction of a hydrophilic surface may compromise membrane toughness in chemical, mechanical, and temperature aspects. To maintain structural stability, inorganic ions can be introduced through intercalation techniques. Additionally, fabricating the membranes as hollow fibers enhances their suitability for water treatment applications. Huang et al. [43] used thermally induced phase separation to prepare PEEK/PEI blended hollow fibers. They employed N-methylpyrrolidone (NMP), methylene chloride, and a composite extractant (NMP/ethanolamine NMP, dichloromethane, and composite extractant (NMP/deionized water) to prepare PEEK hollow fiber membranes. The extraction capacity and crystallization-inducing effect of the three extractants were compared according to the differences in the crystallization-inducing ability of PEEK with different polar solvents. The effects of extraction capacity and solvent-induced crystallization on the membranes’ morphology, pore structure, permeability, and mechanical properties were investigated. Consequently, mechanical strength was decreased while water flux was increased due to the unique characteristics of the hollow structure. In a study by Wang et al. [19], PEEK fibers were prepared through melt spinning and woven into textiles (Figure 1b). These textiles were further transformed into membranes using a one-step reduction method, resulting in highly hydrophilic textiles (PEEK-OH textiles) with excellent melt, selectivity, strength, corrosion resistance, and thermal stability. Owing to the significant voids between the fibers, both PEEK and PEEK-OH textiles contain substantial macropores. In order to utilize the membrane pores effectively, liquid injection was used to regulate the surface energy and hydrophilicity of the PEEK-OH textiles. The separation efficiencies of PEEK and PEEK-OH textiles were evaluated following immersion in various solutions, including 6 mol/L H_2_SO_4_, 6 mol/L NaOH, 6 mol/L NaCl, DMF, and dichloromethane, each for 500 h, respectively. Moreover, the thermal stability of both textiles was assessed after heating at 200 °C. Even after undergoing corrosion treatment, solvent immersion, and heating, the efficiency remains above 99.8%, demonstrating the corrosion resistance and the good thermal stability of PEEK-OH textile membrane materials. Furthermore, Wang et al. [37] developed PPY and polygonal nickel-cobalt LDH nanosheet-modified PEEK textiles, exhibiting double superhydrophobicity and good switching stability (Figure 1c). These membranes demonstrated the ability to separate not only oil/water mixtures but also immiscible organic liquids with different polar components of surface energy (PSE). In addition, due to the “mediator effect” of ethanol, the membrane showed a super high cut-off height for low PSE organic liquids. In another study by Wang et al. [38], ZnO nanopins were successfully immobilized on surface-sulfonated PEEK felts using UV/ozone cleaning and hydrothermal synthesis (Figure 1d). The modified felt (PEEK-f-Z) exhibited better anti-fouling properties and higher rejection height (33 cm) compared to the unmodified felt (17 cm), with a separation efficiency as high as 99.99%. The above-prepared membranes demonstrated good solvent resistance, mechanical strength, and thermal stability, making them suitable for oil/water separation applications in harsh environments.

**Figure 2:**
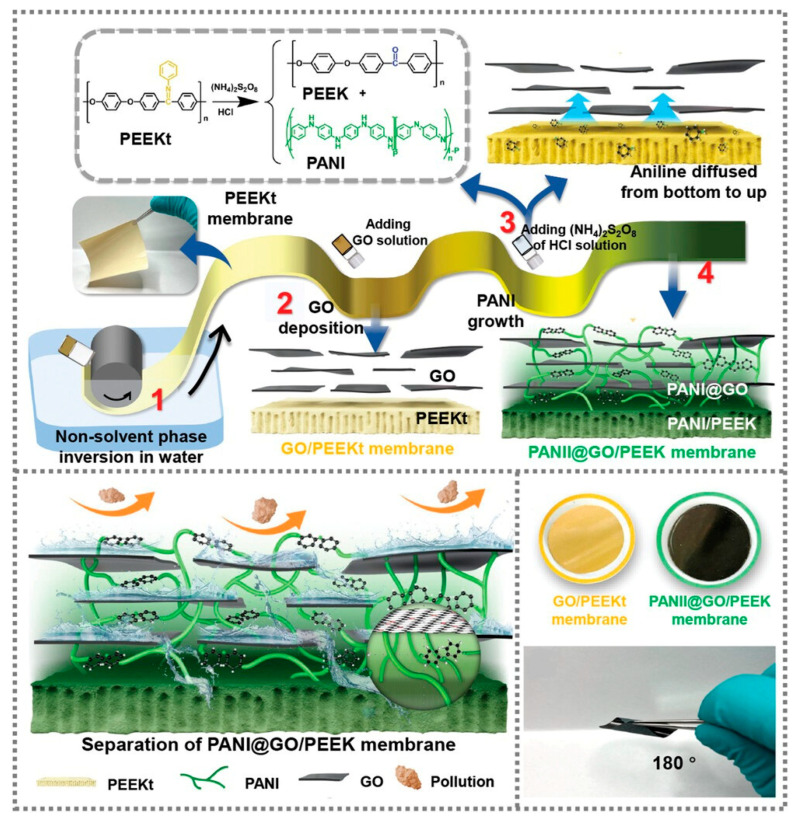
Oxide/polyetheretherketone (PANI@GO/PEEK) membranes are prepared using multifaceted in situ anchoring via PANI [44].

In practical applications, the issue of compatibility between membrane components and composite functional components often persists. To address this, Lin et al. [44] prepared PANI@GO/PEEK membranes using polyaniline (PANI) for achieving multi-facial in situ anchoring. The in-situ growth of polyaniline facilitates the full bonding of the PEEK substrate and the GO separation interface, effectively addressing challenges related to PEEK solution handling and the instability of the GO layer. Employing a bottom-up restricted polymerization approach for aniline enables the control of pore size of the separation layer, correction of defects, and a well-defined relationship between the polymer, the nanoseparation layer, and the anchored nanosheets (Figure 2). The resulting membranes showed exceptional stability under harsh conditions, including exposure to 2 M HCl, NaOH, and high temperatures, with a retention rate exceeding 90%. In addition, the membranes showed remarkable durability after 240 days of immersion and 100 h of long-term operation. This method presents a novel strategy that significantly facilitates the formation of specialized separation membranes.

#### 2.1.2. PEEK Membranes with Anti-Fouling Performance Applied in Complex Environments

**Figure 3:**
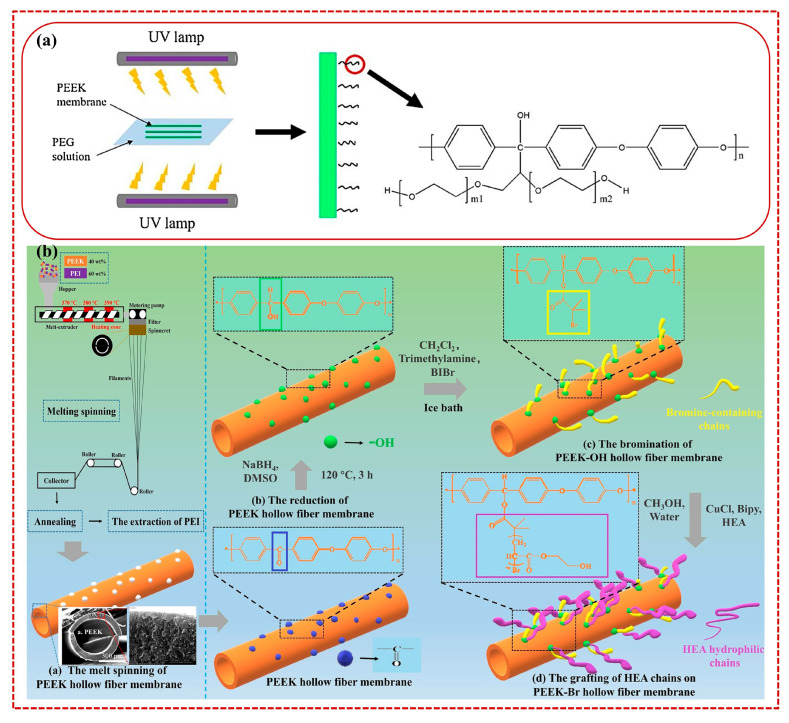
Oxide/polyetheretherketone (PANI@GO/PEEK) membranes prepared using multifaceted in situ anchoring via PANI (**a**) [48] and Polyetherimide (PEI)/PEEK blends melt spinning and PEI extraction for the preparation of PEEK hollow fiber membranes (PHFM) (**b**) [41].

Polymer membranes often suffer from hydrophobic and non-specific interactions between the membrane surface and contaminants, leading to significant membrane contamination [45]. In contrast, advanced PEEK membrane surfaces possess excellent anti-adhesion and anti-pollution properties following purposeful improvement strategies. Enhancing the hydrophilicity of the membrane surface represents one approach to improve its anti-fouling performance. The surface grafting method involves introducing a grafted modified layer on the membrane surface through a chemical reaction, and the modified layer is connected to the membrane surface with a stable covalent bond. The chemical modification only occurs on the surface of the material without affecting the performance of the membrane substrate [46,47]. Therefore, surface chemical grafting enables the attachment of a hydrophilic graft layer onto the membrane surface, thereby enhancing the anti-fouling performance of the membrane. Chen et al. [48] used the UV irradiation grafting method to graft polyethylene glycol (PEG) with a relative molecular mass of 2000, 10,000, 20,000, and 50,000 g/mol onto the surface of the PEEK hollow fiber membrane (Figure 3a). After PEG grafting, the hydrophilicity and permeability of the membrane were improved. However, the longer grafted chains tended to clog the membrane pores. Furthermore, an increase in grafting density (GD) offers dual benefits. On one hand, it prevents dirt from being adsorbed on the membrane surface through the gaps between the chains. On the other hand, it enhances the spatial repulsion of the grafted layer to the dirt. Furthermore, it can increase the distance between the dirt and the membrane base, thus weakening the attraction between the two. Optimizing the GD and chain length of the hydrophilic graft layer can enhance the anti-fouling performance of PEEK hollow fiber membranes. Zhang et al. [41] prepared PEEK hollow fiber membranes (PHFM) by melting spinning poly(etherimide) (PEI)/PEEK blends and subsequently extracting PEI. Then, a hydrated layer consisting of 2-hydroxyethyl acrylate (HEA) chains was constructed on the surface of PHFM using surface-initiated atom transfer radical polymerization (Figure 3b). The grafting density and grafting length of HEA chains were regulated by changing the bromination time and ATRP reaction time, effectively improving the hydrophilicity of the obtained PHFM surface without compromising its original porous structure. Following the modification, the PHEM exhibited a remarkable 90% retention rate of bovine serum albumin (BSA).

#### 2.1.3. PEEK Hollow Fiber Membranes with Salt-Resistant Performance Applied in Harsh Environments

**Figure 4:**
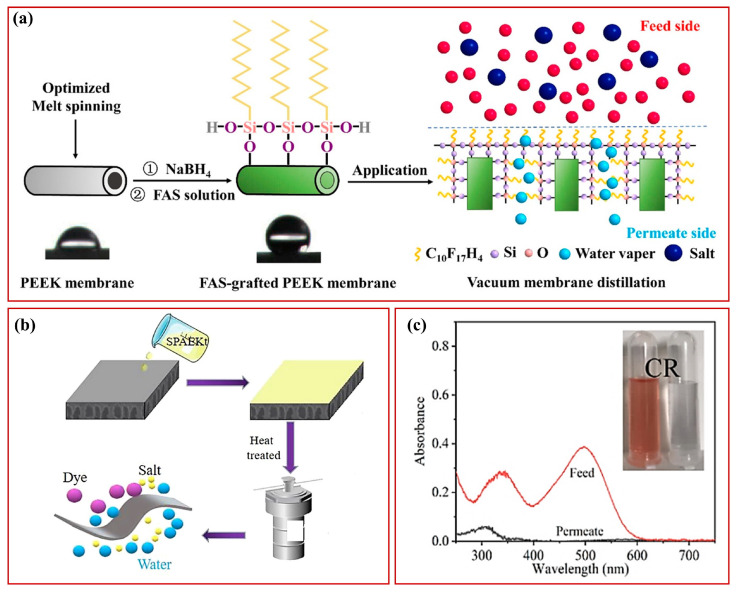
Schematic diagram of silane-grafted hydrophobic PEEK hollow fiber membranes successfully produced using a one-step reduction and silane modification method (**a**) [36], synthesis of sidechain sulfonated poly(ether ether ketone) (SPEEK), and preparation of PEEK-SPEEK nanofiltration membranes using a simple dip-coating and heat treatment (**b**); CR removal rate (**c**) [30].

Many studies reported that PEEK membrane materials can be prepared as adsorbents for the efficient removal of organic dyes in water treatment. Due to its high surface area and good adsorption properties, PEEK membranes hold significant potential for application in treating dye-contaminated wastewater. Furthermore, PEEK exhibits excellent performance even in high-salt environments, demonstrating notable resistance to corrosion caused by salt solutions, seawater, and other salt-containing media. This high performance makes PEEK a promising candidate for a wide range of applications in desalination, seawater treatment, and water treatment in saline environments. The preparation approach of PEEK using amorphous PEEK as raw material was investigated in the study conducted by Song et al. [49]. Sulfonated poly(ether ether ketone) (SPEEK) was coated on poly(ether sulfone) (PES) ultrafiltration hollow fiber membranes to prepare membranes for multifunctional composite hollow fiber nanofiltration membranes for the separation of glyphosate from saline wastewater, and the study adequately verified the salt resistance of PEEK. Zhao et al. [36] used thermally induced phase separation (TIPS) and chemical extraction methods to successfully prepare nanoporous PEEK hollow fiber membranes. Silane-grafted hydrophobic PEEK hollow fiber membranes were successfully prepared using a one-step reduction and silane modification (Figure 4a). The performance of both pristine and modified membranes was investigated for treating a brine feed containing 3.5 wt% NaCl using the vacuum membrane distillation (VMD) process. To achieve efficient oil/water separation and salt resistance, Jin et al. [50] used sulfonated polyether ether ketone (SPEEK) coated commercial polyethersulfone (PES) hollow fibers to prepare membranes. The SPEEK-coated membranes exhibited significantly enhanced oleophobicity compared to the supported PES membranes, possibly due to their non-porous surface, higher hydrophilicity, and a more negatively charged SPEEK surface. However, the water treatment process is also accompanied by dye contamination sometimes. To address this issue, Cao et al. [30] synthesized sidechain SPEEK and prepared PEEK–SPEEK nanofiltration membranes via a simple dip-coating and heat treatment process (Figure 4b). Single-component filtration experiments demonstrated that the optimized membranes had a high pure water flux (126/bar) and a low NaCl retention (6.7%). In contrast, the removal of Congo red (CR) via the negatively charged membrane was 98.8% (Figure 4c). Overall, PEEK demonstrates immense potential for applications in water treatment processes, particularly in harsh environments. Its resistance to acid and alkali, salt resistance, anti-fouling, self-cleaning, and dye adsorption properties make it an ideal high-performance candidate in water treatment areas. With further research and innovation, the application scopes of PEEK in water treatment processes in harsh environments can be further expanded, offering more effective strategies for water treatment challenges.

### 2.2. Polyethersulfone (PES)

**Figure 5:**
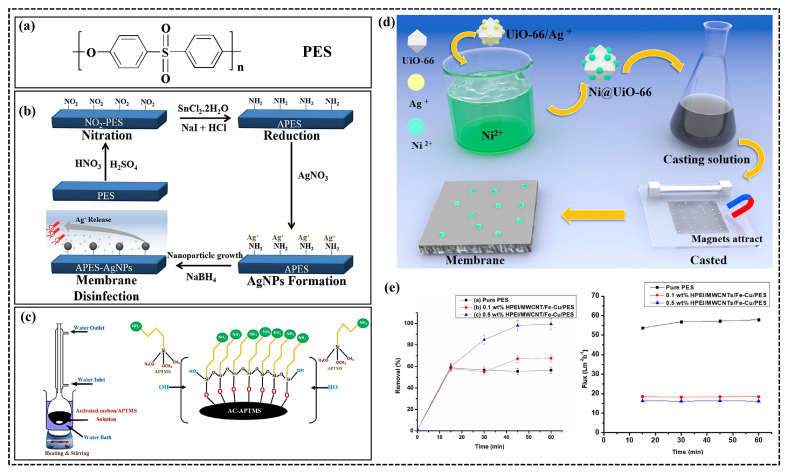
Typical molecular structure of PES (**a**), schematic representation of silver leaching and anti-biofouling effect of Ag NPs-APES samples (**b**) [54], schematic diagram of PES ultrafiltration membrane prepared using the phase change method (**c**) [58], schematic representation of PES-Ni@UiO-66 membranes prepared using an in situ reduction reaction (**d**) [59], pictures of the removal of 2,4,6-TCP via HPEI/MWCNTs/Fe-Cu/PES membranes (**e**) [55].

Polyethersulfone (PES) is a type of thermoplastic polymer synthesized from ether sulfone monomers, primarily comprising sulfone groups, ether groups, and secondary phenyl structures (Figure 5a) [51,52]. The sulfone groups enhance the heat resistance of the polymer, while the ether groups provide good mobility to the polymer chain in the molten state, facilitating ease of formation and processing. Notably, PES demonstrates remarkable temperature resistance, ensuring stable operation in high-temperature environments for extended periods. Furthermore, PES possesses high mechanical strength and rigidity, providing it with the capability to withstand considerable stress and pressure. Its excellent resistance to acids, alkalis, and solvents maintains stability in harsh chemical environments. Additionally, PES delivers commendable separation performance, effectively eliminating suspended solids, colloids, microorganisms, and heavy metal ions from water. Preparing PES in membrane materials can be employed in membrane separation technologies such as ultrafiltration, microfiltration, and nanofiltration, which are crucial in application within seawater desalination, wastewater treatment, and drinking water purification. Moreover, PES can be crafted into filters for solid–liquid separation and particle filtration, widely used in industrial wastewater treatment, food processing, and related fields.

#### 2.2.1. Antimicrobial and Anti-fouling Self-Cleaning of PES Fiber Membranes

In the process of water treatment, membrane fouling, often caused by bacteria and protein contamination, presents a common challenge, significantly impacting membrane reuse efficiency. Hence, membrane materials typically undergo fabrication using methods such as phase transformation [53,54,55] and electrostatic spinning [56]. Followed by functionalization via subsequent chemical modification processes [57]. For example, Shen et al. [53] prepared a series of ZnO/PES hybrid membranes, integrating ZnO nanoparticles via a phase transformation method. After continuous filtration of a BSA solution (1 g/L, pH = 7.4) for 90 min, the flux reduction rate of ZnO/PES hybrid membranes (spiked with 0.3 g of ZnO nanoparticles) was only 7.8%, and the anti-fouling performance was significantly improved compared with that of PES membranes. Furthermore, the introduction of silver nanoparticles onto fibrous membranes imparts long-lasting antimicrobial effects. Otherwise, Haider et al. [54] found that PES, when combined with certain antimicrobial materials, can acquire such properties to give an antimicrobial effect and functionalized PES via a phase transformation method, in which an aminated PES(NH_2_-PES, APES) was formed through involving the introduction of amino groups to form aminated PES (NH_2_-PES, APES). Subsequently, Ag NPs were immobilized on the surface of the APES composite membrane to form Ag NPs-APES, with the systematic investigations of silver leaching and anti-biofouling effects of the Ag NPs-APES samples (Figure 5b). Furthermore, researchers aimed to enhance PES membranes’ functionality by providing anti-fouling protection and imparting antimicrobial properties suitable for more complex water treatment environments. In this pursuit, Abid et al. [58] fabricated PES ultrafiltration membranes through a phase change method using varied concentrations of APTMS (3-Aminopropyltrimthoxysilane) modified activated carbon (mAC) (Figure 5c). The resulting mAC membranes revealed enhanced hydrophilicity, reduced contact angle, improved porosity, roughness, water retention, and heightened water flux. Meanwhile, the mAC composite membranes showed anti-bacterial efficacy against both Gram-negative Escherichia coli (*E. coli*) and Gram-positive Staphylococcus aureus model test strains. The anti-fouling studies using BSA solution filtration demonstrated that the mAC membrane exhibited favorable BSA flux rates.

The combination of PES with metal–organic skeleton materials (MOFs) not only achieve the effect of anti-fouling effects but also exhibits photocatalytic self-cleaning properties. For instance, Liu et al. [59] first encapsulated magnetic Ni on UiO-66 by in situ reduction reaction and then added it into the PES membrane matrix to prepare the PES-Ni@UiO-66 membrane (Figure 5d). The resulting hydrophilic Ni@UiO-66 could be magnetically pulled to the membrane surface, which resulted in high hydrophilicity (WCA 54.5°) and permeability performance. This resulted in the prepared membrane exhibiting a remarkable pure water flux of 611.5 ± 19.8 L/m^2^·h, effectively improving the fouling resistance of the membrane. In addition, using the photocatalytic activity of Ni@UiO-66 exposed on the membrane surface, the obtained PES-Ni@UiO-66 membranes had good photocatalytic self-cleaning ability, with a flux recovery rate (FRR) of more than 95% under UV irradiation. Additionally, composite nanomaterials comprising PES and metal nanoparticles present promising potential for the dichlorination process. Dube et al. [55] employed membranes constructed with highly catalytic Fe-Cu bimetallic nanoparticles, hyperbranched polyethylene imines (HPEIs), and multi-walled carbon nanotubes (MWCNTs) for the degradation of 2,4,6-trichlorophenol, assessing the dichlorination efficiency using liquid chromatography–mass spectrometry (LC-MS) and the Dechlorination efficiency. The FRR of BSA surpassed 90%, and the HPEI/MWCNTs/Fe-Cu/PES membranes achieved up to 99.4% removal of 2,4,6-TCP (Figure 5e), while also enhancing hydrophilicity and permeability. Notably, the study verified the presence of a strong interaction between the nanofiller (HPEI/MWCNTs) and the Fe-Cu bimetallic system.

#### 2.2.2. Other Water Treatment Applications of PES Membranes

PES membranes possess diverse applications such as adsorption of metal ions, salt resistance, removal of dyes, and potential as exchange membranes for selective removal of ions [60,61], making them suitable for treating textile wastewater, food industry wastewater, etc. For example, Giwa et al. [62] utilized electrically enhanced membrane bioreactors (eMBR) to achieve a high-quality effluent at a lower cost compared to conventional methods [63,64]. The eMBR system provided superior removal efficiency of organic matter and water. Furthermore, nucleophilic substituted PES-GO membranes were investigated for treating remediated wastewater obtained after eMBR. All nucleophilic-substituted PES-GO membranes were used to treat remediated wastewater obtained after eMBR. The impact of nucleophilic modifiers (maleic acid, hyperbranched polyethyleneimine, and chitosan) on the performance of GO-assisted hybrid matrix membranes was investigated at a molecular level. The use of PES as a polymer in the hybrid matrix membranes facilitated integral adhesion between GO and PES via the nucleophilic substitution of GO. The overall pollutant removal efficiencies were notable: 97.1% Fe^2+^, 95.3% Zn^2+^, 92.7% Cd^2+^, 99.9% Cr^6+^, and 99.9% bacteria. Marjani et al. [65] experimentally synthesized GO using the modified Hummers method and prepared polymer membranes doped with GO particles via the phase transition method. The incorporation of GO resulted in increased hydrophilicity, leading to improved pure water flux and enhanced salt rejection, particularly at an optimal GO concentration of 3 wt%. This improvement can be attributed to the hydration size of ions transferring through the membrane pores, ion diffusivity, and electrostatic interactions with the membrane. Due to the negative charge on the surface of the prepared nanocomposite membranes and considering the cationic nature of MO and MB and electrostatic repulsion, the retention of MO is greater than MB. Additionally, this PES membrane demonstrated effectiveness in removing Zn^2+^, Cd^2+^, and Cu^2+^ ions, with the highest removal efficiency observed for Zn^2+^ ions.

**Figure 6:**
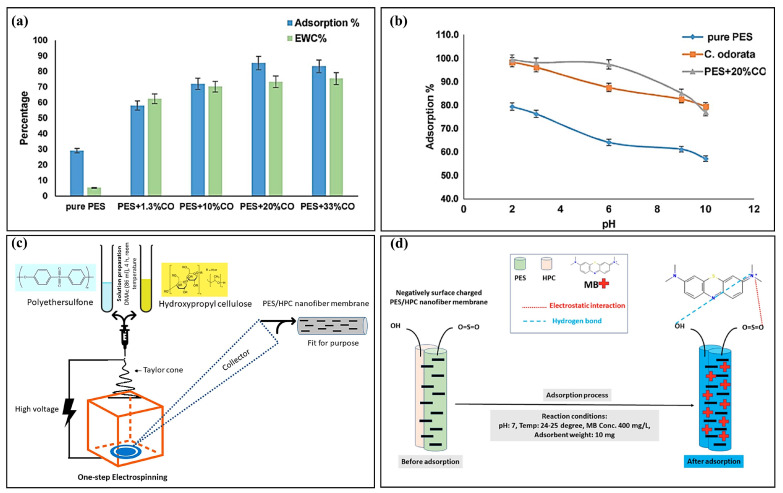
Adsorption effect of PES/CO adsorption film on Congo red dye (**a**), plot of pH on adsorption effect (**b**) [66], schematic diagram of PES/HPC co-blended nanofibrous membrane prepared using the one-step electrostatic spinning method (**c**), and adsorption mechanism diagram (**d**) [56].

PES membrane application for dye adsorption is also a good choice. Zwane et al. [66] developed PES/CO (Polyethersulfone/Chromolaena odorata) adsorbent membranes through the phase transition method to study their potential application in adsorbing Congo red dye. Figure 6a illustrates an increase in the adsorption rate of Congo red on the PES/CO membrane, correlating with an increase in the CO content. However, the adsorption rate began to decrease after reaching a certain ratio, potentially due to the particle agglomeration and subsequent decline in membrane porosity, resulting in reduced available adsorption sites. pH is another important reason affecting the adsorption effect, as shown in Figure 6b. The influence of pH on adsorption was investigated within the range of 2–10, revealing the optimal pH for Congo red adsorption by CO particles, pure PES, and 20% CO-doped PES membranes to be pH 2. At this pH, the respective adsorption efficiencies were 98.4%, 79.4%, and 99.5%. The favorable adsorption at lower pH can be attributed to the increased presence of H^+^ ions, which enhance the number of positively charged adsorption sites, consequently promoting the adsorption of negatively charged Congo red dye molecules. These findings highlight the significance of doped particles and solution pH in determining the adsorption effectiveness of PES membranes during dye adsorption. Nahid Pervez et al. [56] employed a one-step electrostatic spinning process to fabricate a unique polyethersulfone/hydroxypropyl cellulose (PES/HPC) co-blended nanofibrous membrane (Figure 6c), utilizing it for the first time to remove MB from an aqueous solution. Usually, the selected operating parameters, such as initial solution pH, contact time, initial MB concentration, and ionic strength concentration, were carefully investigated to gain insight into the adsorption process, and the preliminary adsorption mechanism is shown in Figure 6d. Sulfonyl functional groups are enriched in the main chain of PES compounds and interact with the cationic MB dye through various forces such as electrostatic interactions, hydrogen bonding, and π–π interactions [67]. The presence of the sulfone group, being more polar than the ether group, accelerates ion exchange, thus enhancing electrostatic attraction and significantly improving the adsorption capacity of PES/HPC nanofibrous membranes [68].

### 2.3. Poly(Arylene Ether Nitrile) (PEN)

**Figure 7:**
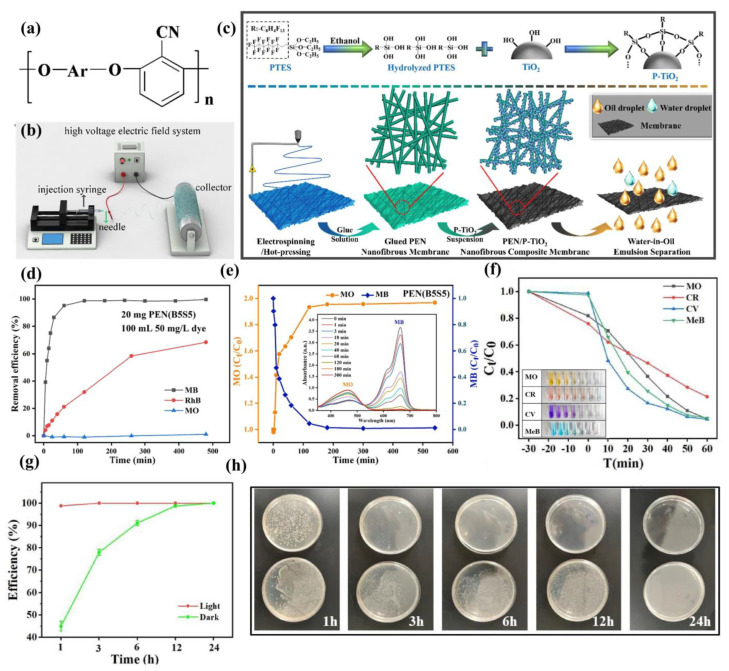
The structural general formula of PEN (**a**). Schematic diagram of electrostatic spinning (**b**) [13]. Preparation process of PEN/P−TiO_2_ nanofiber composite membranes (**c**) [74]. Adsorption effect of Ag@MXene/PEN fiber composite membranes on different dyes (**d**,**e**) [78]. The photocatalytic ability of MO and CV of Ag@MXene/PEN and inhibitory effect on *E. coli* (**f**–**h**) [79].

Poly(arylene ether nitrile) (PEN) is a novel class of PAE macromolecules, see the structural formula in Figure 7a, with outstanding comprehensive properties such as high heat resistance, good strength, strong toughness, and excellent electrical properties [69,70]. In contrast to the other special engineering plastics within the PAEs class, such as PEEK and PES, PEN exhibits distinct characteristics. The presence of strong polar cyano groups on the side chain of PEN minimally impacts the polymer’s fluidity during molding and processing, enabling satisfactory performance [71]. Additionally, these cyano-side groups augment dipole–dipole interactions within the poly(arylene ether nitrile) molecular chain, thereby bolstering the material’s heat resistance and mechanical strength [72], rendering PEN highly promising for applications in the field of water purification. When PEN is employed in water treatment, it is commonly dissolved in organic solvents for electrostatic spinning, according to perverse research. The fundamental principle involves applying an electrostatic field between the injection device and the receiving device (Figure 7b). A jet is formed from the cone end of the spinning solution, subjected to stretching in the electric field, resulting in the acquisition of a nanofibrous membrane of a certain thickness at the receiving device [13,73]. The application of electrostatically spun nanofibrous membranes in water treatment primarily includes membrane separation, adsorption, and photocatalysis. 

In order to cope with the complex water environment, PEN usually introduces different functional groups via polymerization or further composited with some other functional materials and post-modification after being spun into a fibrous membrane, thus achieving the purpose of application in the field of water treatment. For example, He et al. [74] prepared interconnected PEN nanofibrous membrane substrates using electrostatic spinning and hot-pressing techniques. These membranes were subsequently modified with titanium dioxide (TiO_2_) nanoparticles to create superhydrophobic TiO_2_ nanoparticles (P-TiO_2_). Upon this, P-TiO_2_ nanoparticles were compounded onto the surface of PEN nanofibers using a commercial adhesive as a bonding agent to prepare a PEN/P-TiO_2_ nanofiber composite membrane with stable performance, as shown in Figure 7c. This composite membrane effectively separates water-in-oil emulsions even in harsh environments, including strong corrosive solutions, physical damages, and high/low-temperature systems, while sustaining high separation efficiency (separation flux: ~3000 L/m^2^·h, retention >99%) over 10 cycles, and demonstrating robust reusability. 

Furthermore, some textile wastewater is heavily contaminated with dyes. It is interesting to note that the nitrile group in PEN demonstrates high nucleophilicity [75,76], contributing to its inherent affinity for cationic dyes. Studies indicated that sulfonated PEN exhibits substantial adsorption capacity for organic dyes [77]. Li et al. [78] synthesized a series of PENs with different contents of sulfonated groups and prepared nanofiber membranes as substrates for dye adsorption using an electrostatic spinning technique to investigate their efficacy in removing different dyes. The study revealed that the electrospun PEN membrane possessed rapid separation capabilities for cationic organic dyes, with a remarkable adsorption capacity of 796.25 mg/g for methylene blue (MB). Meanwhile, the optimized electrospun membranes exhibited consistent dye removal performance under the influence of varying pH levels and inorganic salt ions. These membranes demonstrated the characteristics of being easy to regenerate and reuse, maintaining a high removal rate of 99% for MB even after undergoing eight separation-regeneration cycles (Figure 7d,e). This study presents a novel approach for developing new nanofiber separation membranes to effectively purify wastewater contaminated with organic dyes. Nevertheless, some soluble organics and microorganisms still contaminate the separation membranes unavoidably, which tends to cause a decrease in membrane flux and service life. Therefore, developing advanced multifunctional separation membranes with high flux and good fouling resistance remains a significant challenge. Feng et al. [79] prepared multifunctional Ag@MXene/PEN fibrous composite membranes by self-assembling Ag@MXene hybrid materials on PEN porous structures. These fibrous membranes were initially obtained through electrostatic spinning and subsequently modified via biologically stimulated dopamine-initiated cross-linking. The inclusion of silver nanoparticles (Ag NPs) facilitated the formation of additional nanoscale channels within the functional layer. Moreover, the cross-linking via dopamine not only contributed to the super-wettability of the membrane surface but also ensured good interfacial interactions between the Ag@MXene hybrids and the corresponding structural stability of the surface. Due to the superhydrophilicity of the composite membrane, its permeability to different oil/water emulsions was as high as 11,957.5 L·m^−2^ h^−1^ bar^−1,^ and the retention rate was as high as 99.13%. Additionally, the Ag@MXene/PEN composite membrane had good photocatalytic ability for methyl orange (MO) and crystal violet (CV) within 60 min. It also exhibited remarkable antimicrobial efficacy, inhibiting *E. coli* growth by ~99.99%, enabling synergistic self-cleaning of the membranes (Figure 7f–h). Apparently, this composite membrane has ultra-high separation flux, excellent retention rate, and unique synergistic anti-fouling ability based on antimicrobial and photocatalytic self-cleaning, providing a new pathway for rapid wastewater purification. Similarly, Sun et al. [80] also achieved synergistic anti-fouling of composite membranes by combining a MXene and ZnO hybrid heterojunction structure with PEN, leading to enhanced antimicrobial and photocatalytic self-cleaning performance of fiber composite membranes for MB degradation. This combination of synergistic anti-fouling and high permeability enables the composite membrane to successfully treat oily wastewater containing multiple pollutants. As a member of the PAEs family, PEN is a relatively novel polymer compared to the above PEEK and PES, with its exploration in water treatment being rare. Based on the existing satisfactory research results, the potential application of PEN in the field of water purification holds significant promise and justifies further development.

## 3. Conclusions and Future Research

This mini-review provides a comprehensive overview of the research status and progress regarding three polymeric materials renowned for their high-temperature resistance and chemical stability: PEEK, PES, and PEN, collectively referred to as typical PAEs. The focus is on their applications in water treatment under harsh environmental conditions. The review begins by delving into the structure, characteristics, and advantages of each polymer, along with their respective application areas. Subsequently, it summarizes the preparation of nanofiber membranes using these polymers via techniques such as electrostatic spinning and phase conversion. Furthermore, the review explores the enhancement of membrane properties through surface modification and the incorporation of nanoparticles, leading to the development of multifunctional composite membranes. These composite membranes not only demonstrate applicability in diverse, complex water environments but also exhibit synergistic anti-fouling and self-cleaning properties.

This review concludes by offering insights into the limitations and future trends in water treatment. It emphasizes the need for addressing the challenges and considerations for further research. Despite considerable progress in developing temperature-resistant polymers and their blends, unresolved issues persist, leaving ample room for advancement in various complex water treatment processes. The subsequent section outlines some of these challenges and proposes potential research directions:

(1) These single polymer composition fibrous membranes are susceptible to membrane fouling and require frequent replacement. While surface functionalization of membranes enhances filtration performance, complex processes hinder industrial product development. Exploring single-step approaches is imperative to enable mass production and simplify the manufacturing process. Consequently, further research is warranted on the production, cleaning, and recycling of membranes;
(2) The fabrication process of certain fibrous membranes often involves hazardous organic solvents. Therefore, conducting research aiming to develop methods for the safe post-processing of fibrous membranes is crucial. The focus lies on converting hazardous substances into useful or dischargeable materials, representing the current research emphasis;
(3) In addition, to maximize the utilization of clean energy and minimize expenses, it is crucial to develop novel fiber membranes that can harness solar energy for efficient and cost-effective water treatment in challenging environments. This endeavor is of paramount importance, striving to achieve high water flux while keeping costs low;
(4) These heat-resistant and stable polymers, combined with nanofibers prepared via electrostatic spinning, serve as excellent materials for water treatment membranes. Their small diameter, porous structure, and large specific surface area make them highly desirable, as they effectively prevent water resource contamination by powders. Consequently, the preparation of nanofibers via electrostatic spinning is currently a crucial research topic in the field of water treatment.

## Data Availability

Not applicable.
